# Natural Variation in *Arabidopsis thaliana* Revealed a Genetic Network Controlling Germination Under Salt Stress

**DOI:** 10.1371/journal.pone.0015198

**Published:** 2010-12-20

**Authors:** Navot Galpaz, Matthieu Reymond

**Affiliations:** 1 Department of Plant Breeding and Genetics, Max Planck Institute for Plant Breeding Research, Köln, Germany; 2 Institut Jean-Pierre Bourgin, UMR1318 INRA-AgroParisTech, INRA Centre de Versailles-Grignon, Versailles, France; United States Department of Agriculture, United States of America

## Abstract

Plant responses to environmental stresses are polygenic and complex traits. In this study quantitative genetics using natural variation in *Arabidopsis thaliana* was used to investigate the genetic architecture of plant responses to salt stress. Eighty seven *A. thaliana* accessions were screened and showed a large variation for root development and seed germination under 125 and 200 mM NaCl, respectively. Twenty two quantitative trait loci for these traits have been detected by phenotyping two recombinants inbred line populations, Sha x Col and Sha x L*er*. Four QTLs controlling germination under salt were detected in the Sha x Col population. Interestingly, only one allelic combination at these four QTLs inhibits germination under salt stress, implying strong epistatic interactions between them. In this interacting context, we confirmed the effect of one QTL by phenotyping selected heterozygous inbred families. We also showed that this QTL is involved in the control of germination under other stress conditions such as KCl, mannitol, cold, glucose and ABA. Our data highlights the presence of a genetic network which consists of four interacting QTLs and controls germination under limiting environmental conditions.

## Introduction

The sessile life style of plants has led to the development of diverse mechanisms required for the perception and response to fluctuating environments. Natural selection under diverse conditions in contrasting ecological niches has lead to enormous genotypic and phenotypic variation in plants. Natural variation was used along the history by farmers to domesticate and further breed for crop plants with desirable traits [1], [2]. Natural variation between and within plant species is also being exploited to reveal the genetic basis of phenotypic variation up to the level of the single polymorphic nucleotide, and emerges as a powerful and complementary approach to the use of artificially induced mutations. Up to now nearly 100 genes and functional alleles underlying natural phenotypic variation have been detected in different plant species [3]–[4].

Wild populations of Arabidopsis, also called accessions, are widespread in the northern hemisphere [5]. Over 6000 *A. thaliana* accessions have been collected [6]. In addition, a large number of mapping populations (F2's, RILs and NILs) was developed (http://dbsgap.versailles.inra.fr/vnat/Fichier_collection/Rech_rils_pop.php) and used for QTL analysis and gene cloning. Natural variation was found for many traits in Arabidopsis, including seed germination and dormancy, flowering time, plant architecture and morphology, growth, primary and secondary metabolism, responses to biotic and abiotic stresses, etc. [3]. Environmental stresses, especially salinity and drought, are the major causes of yield loss worldwide. More than 800 million hectares of land throughout the world, which corresponds to more than 6% of the world's total land area, are salt affected (http://www.fao.org/ag/agl/agll/spush). Different environmental stresses are often interconnected. Thus, drought, salt and extreme temperature stresses cause osmotic and associated oxidative stress, resulting in homeostasis and ion balance modifications in the cell as well as changes in protein and membrane functions [7]–[8]. The plant hormone Abscisic Acid (ABA) is a major player in plant responses to different environmental stresses [9]. Increasing ABA levels in response to environmental stresses drive wide array of adaptive responses including stomata closure, global changes in transcription and the activation of various signal transduction pathways [10].

The mutant approach has led to the identification of several large effect salt tolerance genes including the plasma membrane Na^+^\H^+^ antiporter family member SOS1 [11], the vacuolar Na^+^\H^+^ antiporter NHX1 [12] and a plasma membrane Na^+^ transporter HKT1 [13]. However, the artificial mutant induced approach has a limited potential to uncover the genetic basis of salt tolerance in Arabidopsis: first, the current artificially induced mutant collections are based on a very small number of accessions, mainly Col, L*er* and Ws, representing only a minute fraction of the genetic variation existing in this species. Second, the most common lab strains, Col and L*er*, are relatively salt sensitive [14]–[15], therefore assumed to lack many functional salt tolerance alleles that might be present in other more salt tolerant accessions. Third, plant responses to environmental stresses, including salt, are complex and polygenic traits, and controlled by genetic networks consist of large number of rather small effect loci. The artificially induced mutant approach is suitable mainly for the detection and cloning of large effect genes, but lack the power to detect the ones with small effect as well as the genetic interactions between them. Using natural variation in Arabidopsis is therefore a powerful complementary approach to the artificially induced mutant approach. This approach is based on the identification of the genetic determinism causing phenotypic variation for a given trait between accessions. QTL analysis using mapping populations derived from accessions with contrasting phenotypes allows the identification of this genetic determinism. In previous studies, two screens aiming to detect natural variation in A. thaliana for salt tolerance were carried out, comprising of 102 and 350 accessions [14]–[15]. These studies reported a wide range of salt responses among these sets of accessions. So far, natural variation has been successfully employed to clone two major salt tolerance genes in Arabidopsis; natural alleles of the Na^+^ transporter HKT1, conferring salt tolerance to the coastal accessions Ts-1 (Spain) and Tsu-1 (Japan) were Identified [16]. Recently, QTL analysis using the Sha x L*er* population and subsequent map based cloning led to the isolation of RAS1, a novel negative regulator of salt tolerance [17].

Epistasis, defined as the non-additive relations between alleles at different loci, can expand the range of phenotypic variation in a population. Natural variation in Arabidopsis revealed strong epistatic interactions in diverse traits, including plant-pathogen interactions [18]–[19], metabolic pathways [20] and germination [21].

The ability to germinate under unfavorable conditions is critical for colonizing species like *A. thaliana*, therefore expected to be under strong natural selection.

Unfavorable conditions may result in germination failure or in mortality of the developing seedling. High salt concentrations in the soil reduce water potential and hinder water absorption by the germinating seeds. Penetration of solutes through the seed coat induces osmotic stress and ion toxicity [22].

Natural variation was used for the dissection of the genetic architecture of germination under various environmental stresses in Arabidopsis [14], [23], tomato [24] and barley [25]. Co-location of some of the QTLs detected in these studies suggested common genetic determinants of germination under salt, drought and cold stresses. Co-location of QTLs for ABA and Reactive Oxygen Species (ROS) hinted for a role as common factors controlling germination under environmental stresses [23].

In the present study we screened 87 *A. thaliana* accessions for salt tolerance. The salt sensitive accessions Col and L*er* and the salt tolerant accession Sha were selected to study the genetic basis of germination under salt stress. Although these accessions were able to germinate under 175 Mm NaCl, some RILs in the Sha x Col population failed to germinate under this condition. Interaction between four QTL has been investigated in order to explain the genetic determinism of this particular phenotype. In addition, the effect of one of these QTLs on germination under salt stress has been validated and tested under various stress conditions.

## Results

### Screening *Arabidopsis thaliana* accessions for responses to salt

**Figure 1:**
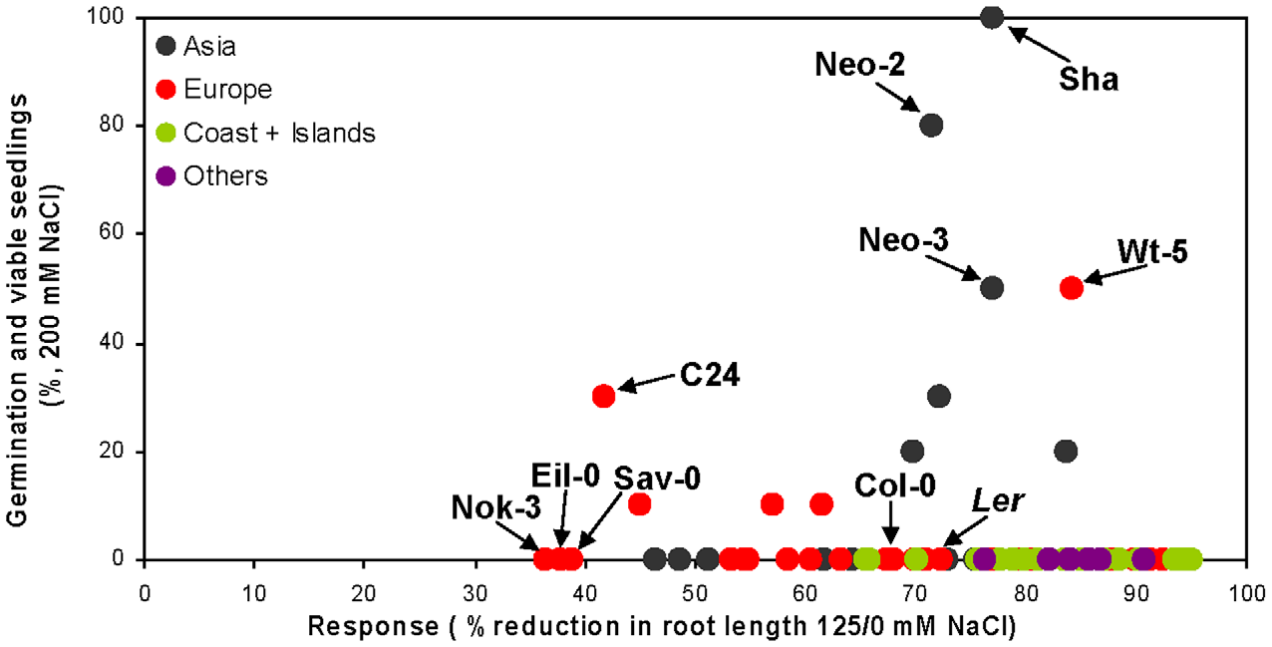
Responses to salt stresses of 87 *Arabidopsis thaliana* accessions. For each accession, the reduction in root length (%) in response to 125 mM NaCl is plotted against the percentage of viable seedlings under 200 mM NaCl. Phenotyping was performed ten days after sowing. Values as well as geographical origin of the accessions are presented in Table S1.

To evaluate the response to salt stress, agar medium supplemented with three different NaCl concentrations (0, 125 and 200 mM) were used. A set of eighty seven accessions (Table S1) that were collected in diverse habitats mainly from Europe and Asia was screened under these salt concentrations. Among them, twenty six accessions were collected in islands or costal habitats in the Netherlands and Germany. These accessions were included in the screen to test whether adaptation to salt tolerance evolved in these presumably more salty environments. Under 125 mM NaCl most of the accessions were able to geminate, but root development was impaired. Seedlings phenotypes of accessions with contrasting responses to salt are presented in figure S1. Thirteen accessions showed extreme sensitivity and failed to germinate in this NaCl concentration although they germinated successfully under 0 mM NaCl (Table S1). The response to salt stress was quantified as the percentage of reduction in root length in 125 mM NaCl compared with 0 mM NaCl (Table S1 and Materials and Methods). Only three accessions: Nok-3 (Noordwijk-3, the Netherlands), Eil-0 (Eilenburg-0, Germany) and Sav-0 (Slavice-0, Czech Republic) showed relatively low response to 125 mM NaCl, manifested by less than 40% reduction in root length (Figure 1). The other accessions were less tolerant, with up to 95% reduction in root length for the island accession OVliel-mw-1 (Oost-Vlmw-1 Middenweg, Netherlands). To evaluate salt tolerance under 200 mM NaCl, the percentage of germinating seeds which managed to develop viable green seedlings four weeks after sowing (seedlings able to withstand and to develop green cotyledons under this conditions) was scored. Sha, Neo-2 and Neo-3 (all three accessions were collected in a close vicinity of the Shakdara river in Tadjikistan, http://dbsgap.versailles.inra.fr/vnat/) and Wt-5 (Wietze-5 from Germany) showed a remarkable tolerance, with 50–100% germination and survival rates, whereas most of the other accessions totally failed to germinate or to develop green viable seedlings under this condition (Figure 1 and Table S1).

### QTL analysis using the Sha x Col and Sha x L*er* populations

Sha, found to be the most tolerant accession under 200 mM NaCl (Figure 1 and Table S1) with 100% germinating seeds and viable green seedlings, was previously crossed to other accessions in order to develop RIL populations (for details see Table S2): Sha x L*er* (Landsberg *erecta* from Poland; [23] and Sha x Col (Columbia from Poland; [26]). By contrast to Sha, L*er* and Col are salt sensitive accessions (Figure 1 and Table S1). We used these two mapping populations to dissect the genetic architecture of salt tolerance in *A. thaliana*. Sets of RILs (see Materials and Methods) from each population were sown on agar medium supplemented with 0, 125 and 175 mM NaCl.

**Figure 2:**
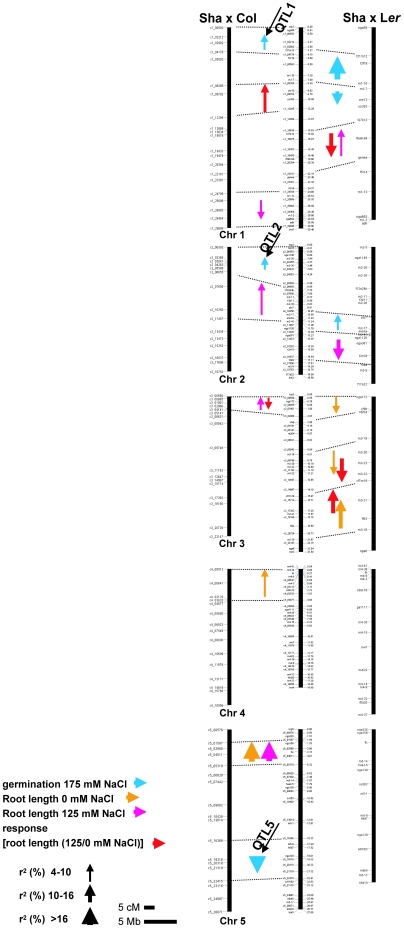
QTL detection in Sha x Col and Sha x *Ler* RIL populations. QTLs for the traits analyzed were mapped on the physical and genetic maps of each population. The 5 Chromosomes of *A. thaliana* genome are represented by vertical bars. Marker names are indicated on the left of each chromosome in the genetic maps of Sha x Col (left) and Sha x L*er* (right). Marker name and physical position (in Mb) are indicated on the physical map (middle). Dashed lines link the physical and genetic positions of the markers flanking the detected QTLs. QTL1, QTL2 and QTL5 involved in the variation of germination under salt stress are indicated on the Sha x Col genetic map. Arrows are color-coded depending on the trait and their length depicts 2 LOD support confidence interval. Arrowheads pointing up indicate that Sha alleles increased trait value. Arrow width indicates r^2^ range (% of explained variance). Phenotyping was performed ten days after sowing.

Four traits were scored; root length in 0 and 125 mM NaCl, response (% of reduction of root length in 125 mM NaCl compared with 0 mM NaCl, see Materials and Methods) and germination under 175 mM NaCl. QTLs involved in the variation of all traits were detected (Figure 2 and Table S3).

Five and six QTLs were detected for root length in 0 and 125 mM NaCl in the two RIL populations, respectively. Heritabilities for both traits were high, ranging from 0.76 to 0.85. Five QTLs were detected for the response of root length to salt (Figure 2 and Table S3).

Under 175 mM NaCl, seeds for which the radical had emerged through the seed coat were considered as geminated. Heritabilities for this trait were 0.65 (Sha x L*er*) and 0.95 (Sha x Col). In the Sha x L*er* population, two QTLs on chromosomes 1 and one on chromosome 2 explaining in total 30.4% of the observed variance were detected (Figure 2 and Table S3). The two linked QTLs on chromosome 1 have opposite effects. Sha alleles on chromosome 2 increased germination rate under salt stress. In the Sha x Col population, QTLs on chromosomes 1, 2, and 5 were detected. Sha alleles on chromosomes 1 and 2 increased germination rate at 175 mM NaCl, whereas Sha alleles on the bottom of chromosome 5 decreased the value of this trait. All together, these QTLs explained 29.8% of the phenotypic variance. (Figure 2 and Table S3).

### Genetic interactions between four QTLs control germination under 175 mM NaCl in the Sha x Col population

During the phenotyping of the Sha x Col population under 175 mM NaCl, a strong transgression was observed, as some of the RILs failed to germinate under this condition, although both parental lines were able to do so (Figure S2). Seedlings of the salt sensitive parental line Col underwent rapid bleaching and died shortly after germination, whereas Sha seedlings showed high tolerance and maintained green and viable seedlings (Figure S3).

**Figure 3:**
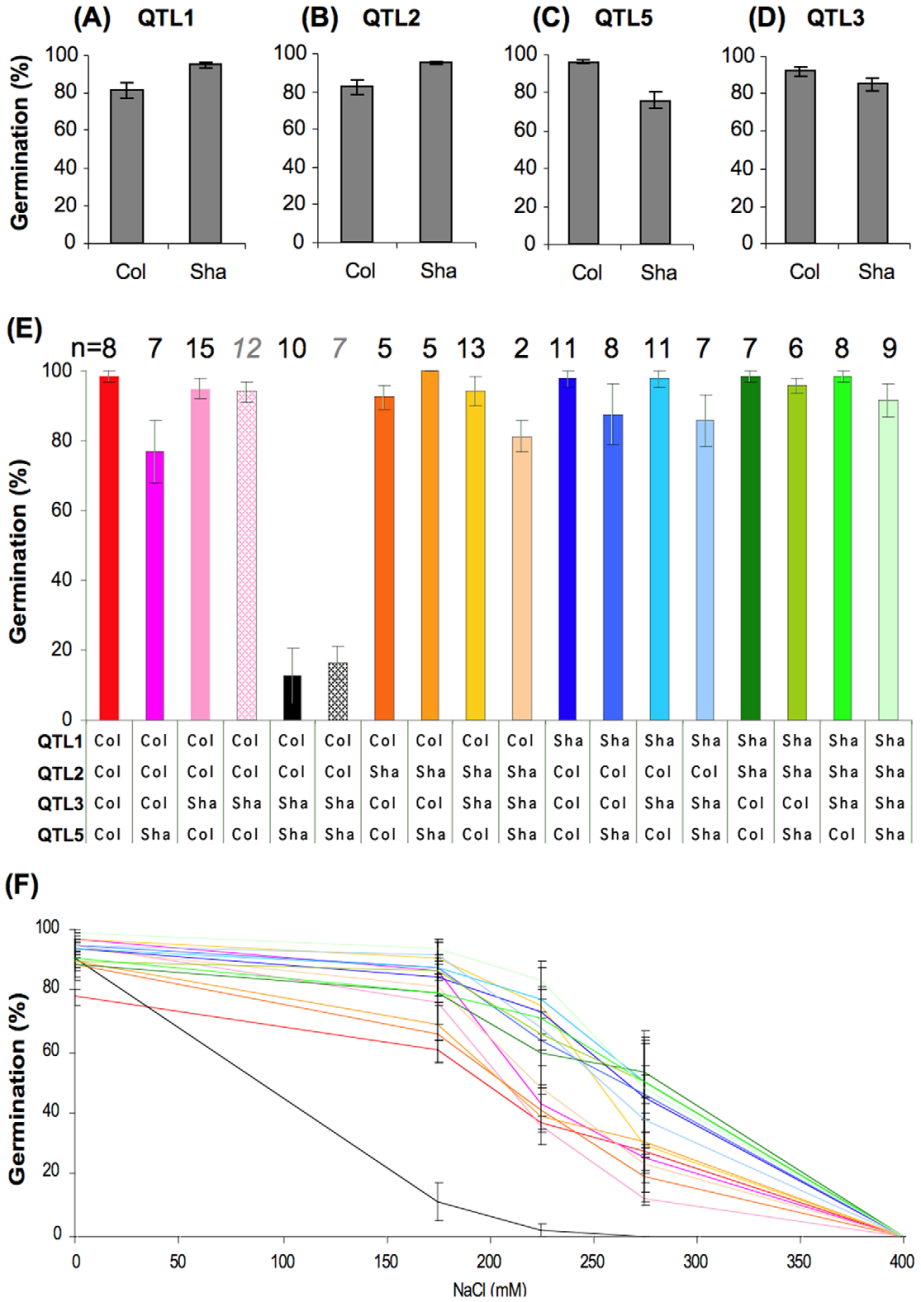
Effect of QTL1, QTL2 QTL3, QTL5 and the effect of their genetic interactions on germination under salt stress. (**A–D**) RILs were classified according to their allelic condition (Sha or Col) at each of the detected QTLs for germination under 175 NaCl, and average germination rate was calculated for each genotypic class. (**E**) RILs were classified according to their allelic combination at the four QTLs and average germination rate under 175 mM NaCl was calculated for each genotypic class. Dotted bars depict the results from an independent experiment using different subset of 19 RILs. n = number of RILs of each genotypic class. Data are means ± SE. (**F**) Four RILs from each of the sixteen allelic combinations at the four QTLs were used for germination test under 0, 175, 225, 275 and 400 mM NaCl. Each of the 16 allelic combinations has the same color code in Figure 3E and 3F. Data are means ± SE.

QTL analysis detected three QTLs affecting germination under salt stress in the Sha x Col population. Col alleles at QTL1 (chromosome 1, marker c1_02992, 7.2 cM) and QTL2 (chromosome 2, marker c2_04263, 9.3 cM) decreased germination rate, whereas Sha alleles at QTL5 (chromosome 5, marker c5_20318, 63.6 cM) reduced germination rate under 175 mM NaCl (Figure 2 and Table S3). To graphically represent the effect of each QTL, all lines from the Sha x Col population used for QTL analysis were classified into two classes according to their allelic condition at each of the detected QTLs, and average germination rate under 175 mM NaCl in each genotypic class was determined (Figure 3A–C). The effect of each QTL on the trait is relatively small and none of these QTL *per se* can explain the no-germination phenotype observed for some of the RILs. The contribution of QTLs with negative effect on germination rate by both parental lines (Figures 2, 3A–C and Table S3) can explain partly this transgression, but the sum of the additive effects of these QTLs cannot explain the no-germination phenotype observed in this population. We then reasoned that at least one more locus affecting germination under salt stress could be present, but might not have been detected during the QTL analysis. It is worth to note that a putative but not significant QTL located on chromosome 3 (with LOD score of 1.8, marker position c3_15714, 41 cM) was detected during the QTL mapping (Figure S4).

The QTL detection has been performed on the core set of the Sha x Col population, which consists of 164 lines, whereas the full set consists of 346 RILs (see Materials and Methods and http://dbsgap.versailles.inra.fr/vnat/Documentation/13/DOC.html). to test the presence of additional loci that might be involved in the no-germination phenotype observed in some of the RILs, we selected from the full set of the Sha x Col population all RILs, 19 in total, carrying the allelic combination leading to reduced germination rate under 175 mM NaCl (Col alleles at QTL1 and QTL2 and Sha alleles at QTL5), and their germination under 175 mM NaCl was scored. Variation for germination rate was still observed among these selected RILs and some of them failed to germinate. In order to uncover the additional locus/i underlying this phenotypic variation, a student test was performed for each marker over this subset of 19 RILs. This analysis revealed that the allelic variation at the interval between markers c3_12647 and c3_17283 (38.7–41 cM) on chromosome 3 can significantly explain the observed variation in germination (P_value_ = 0.05, Figure S5) in these 19 selected RILs. This interval overlaps with the position of the putative QTL that has been suggested during QTL analysis (Figure S4).

Re-analysis of the data used for QTL analysis confirmed that the Sha alleles at this locus, designated as QTL3, decreased germination rate under 175 mM NaCl (Figure 3D). However, the sum of the effects of these four detected QTLs still cannot explain the observed no-germination phenotype. We then investigated if a specific allelic combination at these four loci can explain this phenotype. For that purpose, the set of RILs that was phenotyped for QTL analysis was split into 16 classes according to their allelic condition at the four detected QTLs, and average germination rate for each class was determined. Only the allelic combination which consists of RILs carrying the Col alleles at QTL1 and QTL2 and Sha alleles at QTL3 and QTL5 showed a remarkable inhibition of germination under 175 mM NaCl, whereas all the other allelic combinations showed normal germination rate (Figure 3E). These results strongly suggested that the inhibition of germination was caused by epistatic interactions between the four QTLs.

ANOVA analysis has been performed using models either including only the effect of each QTL (Table S4A) or including the effect of the epistatic interaction between them (Table S4B). When the epistatic interaction was added, all the terms became more significant, the total variance of the model increased (from 33% to 68%) and the term for epistatic interaction explained more than 50% (p_value_<0.0001) of the variation of germination under 175 mM NaCl. This result reinforces the presence of the epistatic interaction between the QTLs in the genetic network controlling germination under salt stress.

We also investigated the effect of each QTL for their response to higher NaCl concentration by phenotyping 4 RILs from each of the 16 genotypic classes at the 4 QTLs (Figure 3F). In this way we addressed the question whether the observed epistatic interaction at 175 mM NaCl holds true over a wide range of NaCl concentrations or results from additive effects where the inhibition curves of the different genotypes gradually shift to higher NaCl concentrations but still germinate almost 100% at 175 mM. In agreement with the results of the QTL analysis, the salt sensitive allelic combination (Col alleles at QTL1 and QTL2 and Sha alleles at QTL3 and QTL5) showed a significant reduced germination rate in all NaCl concentrations tested (Figure 3F). Only for QTL1 a limited main effect was observed, as the RILs carrying Col alleles at QTL1 responded more to NaCl than the ones carrying the Sha alleles at this locus (Figure 3F). Therefore, we concluded that the no-germination phenotype observed in the Sha x Col population is due to a specific allelic combination at the four QTLs in a wide range of NaCl concentrations.

### Validation of the effect of QTL5 on germination under 175 mM NaCl

**Figure 4:**
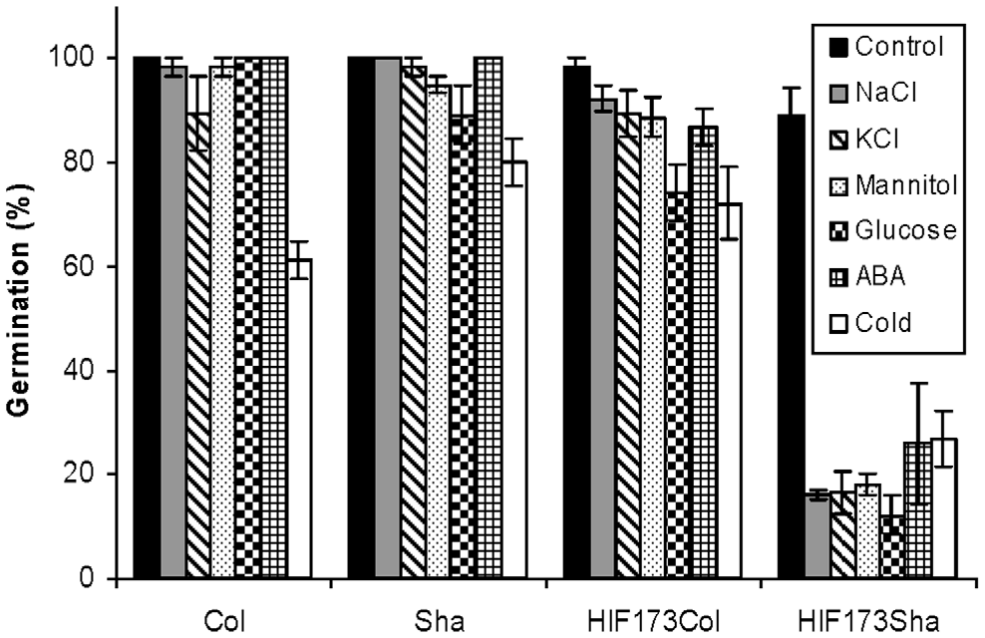
Validation of the effect of QTL5 on germination under salt and various environmental stresses. Germination rate of parental accessions and selected HIF173_Sha_ and HIF173_Col_ lines was scored under the following conditions: 125 mM NaCl, 130 mM KCl, 320 mM Mannitol, 6% glucose, 2.5 µM ABA and 4°C 10 days after sowing. Data are means of four independent lines ± SE.

QTL5 has the largest effect on germination under salt stress in the Sha x Col population, especially in the specific genetic background described above (Figure 3A–D and Tables S3 and S4A, B). To validate the effect of this QTL, a significant variation in germination under salt stress should be observed between two isogenic lines segregating only at the genomic region harboring the QTL. To do so, HIF lines [27] were selected. Because of the observed epistasis involving this QTL in response to NaCl (Figure 3E), the HIF line should segregate at QTL5 and carry the no-germination allelic combination at the other QTLs (i.e. Col alleles at QTL1 and QTL2 and Sha alleles at QTL3). RIL173 fulfils these requirements (Figure S6) and HIF from the progeny of this RIL were selected: HIF173_Sha_ (Sha alleles present at QTL5) and HIF173_Col_ (Col alleles at QTL5). Germination of these lines was nearly 100% in control agar medium without NaCl whereas HIF173_Sha_ seeds showed very low salt tolerance, with 14% germination compared to 97% germination of HIF173_Col_, already under 125 mM NaCl (Figure 4). This result confirmed the effect of QTL5 on germination under salt stress.

### QTL5 controls germination under various environmental stresses

**Figure 5:**
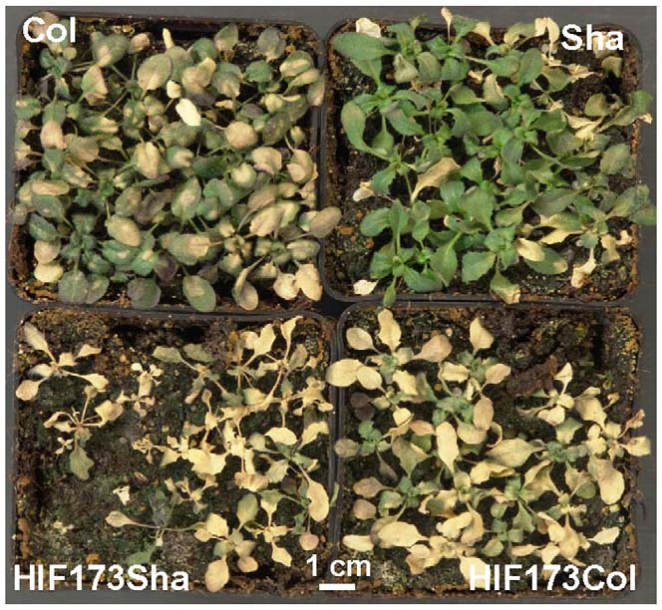
Salt tolerance of HIF173 lines developing seedlings. Three week old plants were watered with 400 mM NaCl twice a week for five weeks.

We then quantified germination of the HIF173 lines (graphical representation of the genotype of these lines is presented in Figure S6) under various environmental stresses in order to further characterize the effect of QTL5. Seeds of the HIFs were sown on agar medium supplemented with NaCl, mannitol, KCl, glucose and ABA, and germination rate was determined. To test whether inhibition of germination under environmental stresses can also be observed in more natural conditions, germination was also scored in soil pots saturated with 300 mM NaCl or transferred to 4°C immediately after sowing. Failure of HIF173_Sha_ seeds to geminate on agar medium supplemented with 125 mM NaCl or 130 mM KCl, by contrast to normal germination of HIF173_Col_ seeds (Figure 4) implied that QTL5 controls the response to different ions and not only sodium. Mannitol has been described to induce osmotic stress [28]. The inhibition of germination of HIF173_Sha_ but not HIF173_Col_ seeds on agar medium supplemented with 320 mM mannitol suggests that QTL5 controls the response to osmotic stress during germination. QTL5 effect is mediated by ABA, as reduced germination of HIF173_Sha_ but not of HIF173_Col_ seeds was observed in the presence of 2.5 µM ABA (Figure 4). High levels of sugars inhibit germination [29]. The failure of HIF173_Sha_ seeds to germinate under 6% glucose indicates for a role of QTL5 in sugar signaling during germination (Figure 4). HIF173_Sha_ lines showed also sensitivity for germination in saline soil. Only 36% of HIF173_Sha_ seeds managed to germinate in soil pots that were watered with 300 mM NaCl, compared with 75% germination in HIF173_Col_ (data not shown). Germination rate of HIF173_Sha_ seeds at 4°C was very low (26%) compared with that of HIF173_Col_ seeds (72%, Figure 4). Taken together, these results indicate that in the context of the revealed genetic network, QTL5 controls germination under a wide range of environmental stresses. To test whether QTL5 controls also the response to salt stress in developing seedlings, three week old seedlings were treated with 400 mM NaCl. Five weeks later Sha plants survived and maintained green color, Col plants showed increased chlorosis and both HIF173_Col_ and HIF173_Sha_ plants died (Figure 5), suggesting that QTL5 is not involved in the response to salt stress in later stages of plant development. Exposure of seedlings to lower NaCl concentrations (100 and 200 mM) results in growth inhibition. However, no differences in growth inhibition were observed between HIF173_Col_ and HIF173_Sha_ plants (data not shown).

## Discussion

We have harnessed natural variation to study the genetic basis of salt tolerance in *A. thaliana*. Wide variation was observed between eighty seven accessions for the reduction of root length in response to 125 mM NaCl and percentage of germinating seeds which developed viable seedlings under 200 mM NaCl. The accessions that showed high tolerance to moderate salt levels (125 mM NaCl) are sensitive to severe salt stress (200 mM NaCl) and vice versa (Figure 1 and Table S1), suggesting that the genetic basis of the response to various levels of salt stresses is different. Similar observation was reported previously [14]. In this study, salt tolerance of 102. *A. thaliana* accessions was quantified, and the most tolerant accessions at the germination level (under 250 mM NaCl) were the most sensitive at the seedling growth level (under 50 mM NaCl). Similar results, indicating separated genetic bases for germination and seedling growth under different levels of salt stresses, were obtained for other plant species; tomato [30], barley [25] and alfalfa [31]. Interestingly, local adaptation to life near the sea was not revealed in our screen, as none of the island or coastal accessions showed tolerance to the applied salt concentrations (Figure 1 and Table S1).

We then used two RIL populations (Sha x Col and Sha x L*er*) for QTL analysis. Reduction in root length in response to 125 mM NaCl and germination rate under 175 mM NaCl were scored. In both populations different sets of QTLs were detected for each of the traits (Figure 2 and Table S3). This observation reinforces the conclusion that the response to different levels of salt concentrations at different developmental stages is driven by distinct genetic determinants. Comparison of the physical positions of the QTLs that were detected in both populations, having Sha as a common parent, revealed that only one QTL co-localizes between the two populations, on top chromosome 3 (Figure 2), suggesting that the sensitivity to salt observed in Col and L*er* accessions is also governed by different genetic determinants.

Interestingly, although Sha, Col and L*er* did not differ much for the reduction of root length in response to 125 mM NaCl (Figures 1 and S3), three QTLs, explained 26.2% of the variation (Sha x L*er*), and two QTLs, explained 15.5% of the variation (Sha x Col) were detected for this trait (Figure 2 and Table S3). The uncovering of salt tolerance QTLs controlling root development under salt stress might be explained by the presence of positive and negative alleles at different loci in the different parents. Four QTLs controlling germination under 175 mM NaCl were detected in the Sha x Col population (Figures 2, 3A–D and Table S3). QTLs controlling germination and growth under salt stress were detected in various RIL populations in previous studies; Col x L*er* [14], Sha x L*er* [17], [23], Sha x Bay-0 [32] and Bu-5 x Col [15]. All these populations share at least one parental line with the accessions used in this study. Some of these QTLs were mapped to similar positions with the germination QTLs detected in this study in the Sha x Col population. Salt stress response QTLs were detected on chromosome 1 in the Sha x L*er*, Sha x Bay-0 and Col x L*er*. These QTLs are located in a similar region as QTL1 (marker c1_02992, confidence interval 4–10 cM). Recently the gene *RAS1*, a negative regulator of salt tolerance during seed germination and early seedling growth, was cloned from the Sha x L*er* population [17]. Similarly to QTL1, the Sha alleles of *RAS1* increased salt tolerance. The similar function and location suggest that *RAS1* is underlying the effect of QTL1. A QTL for germination under salt stress was also previously detected in the Col x L*er* population on chromosome 2 and is co-localized with QTL2 (marker c2_04263, confidence interval 7.2–11.3 cM). QTLs controlling germination under salt stress detected on chromosome 3 in the Col x L*er*, Sha x Bay-0 and Sha x L*er* populations co-localize with QTL3 (interval between markers c3_12647-c3_17283, 38.7–41 cM). Salt stress QTLs with a similar position to QTL5 on chromosome 5 (Marker c5_20318, confidence interval 62–66 cM) were detected in the Sha x L*er*, Sha x Bay-0, Col x L*er* and Bu-5 x Col populations. However, no epistatic interactions were reported in the previous studies mentioned above between these QTLs.

In the present study we describe the detection of four loci that are interacting to control germination under salt stress. However, various levels of sensitivity to salt stress were found among the different RILs carrying the no-germination allelic combination (Col alleles at QTL1 and QTL2 and Sha alleles at QTL3 and QTL5). Thus, HIF173_Sha_ showed reduced germination already at 125 mM NaCl (Figure 4), whereas most of the RILs carrying the no-germination allelic combination germinate normally under 125 mM NaCl and show salt sensitivity only at 175 mM NaCl (Figure 3E and data not shown). These differences in the levels of salt sensitivity must be explained by the presence of additional locus/loci that is/are contributing to the response to salt stress in the Sha x Col population, as these lines carry identical allelic combination at the four detected QTLs, but each of them carries a specific genetic background with alleles from Sha or Col at each locus.

To further study the mode of action of QTL5 we have scored germination rate of HIF173 lines (graphical presentation of their genotypes presented in Figure S6) under a set of environmental stress conditions. NaCl causes damage to the plant cell mainly by the induction of osmotic stress and ion toxicity [33]–[34]. HIF173_Sha_ lines showed reduced germination rate compared with HIF173_Col_ lines in the presence of NaCl, KCl, and Mannitol (a common inducer of osmotic stress; Figure 4). These results suggest that the effect of QTL5 is not restricted to sodium detoxification but that QTL5 is rather involved in the response to osmotic stress. The fact that the differences in germination rate between HIF173 lines were observed short time after the exposure to salt stress supports the conclusion that QTL5 is likely controls mainly the response to osmotic stress, which occurred short time after the exposure to NaCl, whereas the buildup of an ionic stress is much slower and take days to weeks [34]. QTL5 is also involved in the control of germination under cold stress, as revealed by the remarkable reduction in germination rate at 4°C of HIF173_Sha_ seeds compared with HIF173_Col_ seeds (Figure 4). HIF173_Sha_ seeds also showed germination sensitivity to glucose (Figure 4), suggesting that QTL5 is involved in the control of sugar signaling during seed germination. It is important to note that not only on agar medium, but also under soil saturated with 300 mM NaCl, HIF173_Sha_ seeds showed reduced germination rate compared with HIF173_Col_ seeds. It will be of interest to test in the future the effect of QTL5 on germination under more natural conditions such as soil watered with lower NaCl concentrations or subjected to different combinations of stress conditions (e.g salt and drought stress, etc).

The involvement of QTL5 in the response to different environmental stresses is in line with previous studies showing a common genetic control of germination under various environmental stresses. Indeed, QTL analysis of germination under salt, drought and cold stresses in tomato revealed that 71% of the detected QTLs affected germination under two stresses or more [24]. In addition, overexpression of a high mobility group B (CsHMGB) protein in Arabidopsis results in the inhibition of germination under salt and drought stresses [35].

A possible link for the control of germination under different environmental stresses is the plant hormone ABA, a key component in dormancy and germination in response to different environmental stresses [9]. ABA is also inter-connected with sugar signaling during seed germination [29]. The inhibition of germination of HIF173_Sha_ seeds in agar medium supplemented with ABA (Figure 4) suggests that QTL5 function is indeed mediated by ABA. In addition to QTL5, also the function of QTL1 which is co-localized with *RAS1*, is mediated by ABA [17], supporting the suggested role of ABA in the regulation of the genetic network revealed in this work. The role of ABA in the control of germination under salt stress was demonstrated by the ability of ABA deficiency and insensitivity mutants to germinate in presence of increased NaCl levels [36]–[37].

Transgression, defined as the formation of extreme phenotypes observed in segregating hybrid populations when compared to parental lines [38], was observed in this study as some of the RILs in the Sha x Col population failed to germinate under 175 mM NaCl, although both parental lines were able to do so (Figures S2 and S3). The observed transgression can be explained by the contribution of salt sensitive alleles from each of the parental lines (Figure 3A–D).

Strong epistasis in the genetic network controlling germination under salt stress was revealed in this study, as only one specific allelic combination of the four detected QTLs results in the inhibition of germination under 175 mM NaCl in the Sha x Col population (Figure 3E). Germination test under different concentrations of NaCl revealed a limited additive effect of QTL1 on germination under NaCl concentrations higher than 175 mM (Figure 3F). The other QTLs in the genetic network do not show any effect *per se* but epistatically interact as only one specific combination of the three QTLs with Col alleles at QTL1 lead to repression of germination under salt stress (Figure 3E, F). The effect of QTL1 is most probably due to the presence of a gene encoding for *RAS1*, a putative transcription factor [17]. Thus, this locus might function as the genetic switch of the genetic network revealed in this work by regulating the expression of the other QTLs, to control germination under salt.

Cloning of the QTLs will shed light on the molecular mechanisms governing the response to salt stress driven by the genetic network revealed in this study.

## Materials and Methods

### Plant material and growth conditions

Eighty seven *A. thaliana* accessions available at the Max-Planck Institute for Plant Breeding Research were screened for salt tolerance (Table S1). The core sets of the recombinant inbred line (RIL) populations Sha x Col-0 and Sha x L*er* (Table S2) were used for QTL analysis. Subset of 19 RILs from the full set of the Sha x Col population (http://dbsgap.versailles.inra.fr/vnat/Fichier_collection/Rech_rils_pop.php) was used for the detection of QTL3. HIFs (Heterozygous Inbred Families) were selected from progeny of RIL 173 (Sha x Col population, Figure S6) for the validation of the effect of QTL5. In addition, 64 RILs from the Sha x Col population were selected according to their genotype at the four detected QTLs controlling germination under salt stress (n = 4 per allelic combination) and their germination was scored under 0, 175, 225, 275 and 400 mM NaCl.

For agar medium experiments, seeds were sown on 12×12 cm plates containing 1% agar medium supplemented with 0.44% MS [39] and 1% sucrose. Plates were incubated at 4°C during four days after sowing and placed vertically in a growth chamber (22°C, 12-h day period). To test germination under various environmental stresses, agar medium was supplemented with 125, 175 or 200 mM NaCl, 320 mM mannitol, 130 mM KCl, 6% glucose or 2.5 µM ABA. To test germination in the soil, seeds were placed on filter paper supplemented with 800 ml tap water and incubated at 4°C for four days and then sown in 7×7×7 cm pots containing Mini-Tray soil (Balster Einheitserdewerk, Germany). Pots were transferred to growth chambers (4°C or 22°C, 12-h day period) and germination was scored ten days after sowing. To evaluate growth and survival of developing plants in response to salt stress, three week old seedlings were watered with 100, 200 and 400 mM NaCl twice a week for five weeks.

### Quantification of germination rate, root length and survival

Germination was scored ten days after sowing in all experiments, directly from the agar medium or the soil. Seeds for which the radical (in the agar medium experiments) or cotyledon (in the soil experiments) had emerged through the seed coat were considered as geminated. In all germination experiments each line was replicated three times and 30–50 seeds were used per replica. To avoid position effect, each replica was placed in different plate and was placed in a different position on the plate and plates were rotated every day. Lines in which germination rate was lower than 80% in the control (0 mM NaCl) agar medium or soil, indicating low seed quality, were excluded from the analysis. For each replica, percentage of germinating seeds was determined and mean of the three replicates was then calculated. For root length measurement, plates were scanned and root length was measured 10 days after sowing using the software ImageJ (http://rsbweb.nih.gov/ij/). Response to 125 mM NaCl, manifested by reduction of root length in 125 mM NaCl compared with 0 mM NaCl medium was calculated as followed: [root length ((0 mM NaCl–125 mM NaCl)/0 mM NaCl)*100)]. For the scoring of survival under 200 mM NaCl, number of germinating seeds which developed green seedlings was counted 10 days after sowing.

### QTL detection and epistasis analysis

MapQTL (version 5.0, Kyazma BV, http://kyazma.nl/) was used to identify and map QTLs using both interval mapping and multiple-QTL model mapping (MQM) methods as described [40]. The estimated additive effect and the percentage of variance explained by each QTL as well as the total variance explained by all the QTLs affecting a trait were obtained for the final MQM model. The cofactors used in the final MQM models are markers around a putative QTL position which are maximizing the LOD score. Permutation test was performed for each trait and each RIL population (n = 1000 repetitions) to determine LOD threshold of QTL detection. LOD values corresponding to P = 0.05 varied between 2.4 and 2.6 depending on the trait under study. 2-LOD support intervals were established as 95% confidence intervals [41]. For the detection of QTL3, the statistical package SPSS 13.0 for windows (SPSS) has been used to perform student test marker per marker over a subset of 19 RILs carrying the salt sensitive allelic combination (Col alleles at QTL1 and QTL2 and Sha alleles at QTL5).

SPSS 13.0 has been also used to perform analyses of variance (ANOVA) to test the significance of main effects and interactions between the QTLs involved in the variation of germination rate under salt in the Sha x Col population. Heritability, defined as the proportion of phenotypic variation that is attributable to genetic variation among individuals compared to the total variation observed, was calculated using the general linear model module of SPSS 13.0.

## Supporting Information

Figure S1 **Contrasting responses of four** ***Arabidopsis thaliana*** **accessions to different salt concentrations.** Pictures of 10 days old seedlings from four *Arabidopsis thaliana* accessions (Col, Sav-0, Kyo and Sha – Supporting Information Table S1) grown in agar medium supplemented with 0, 125 and 200 mM NaCl.
(TIF)

Figure S2 **Graphical representation of the transgression for germination under salt stress in the Sha x Col RIL population.** Percentage of lines (for the RILs) or values from different experiments (for the parental lines) was plotted against percentage of germination under 175 mM NaCl in each genotypic group. n = 12 different experiments for the parental lines, 9 lines for RILs carrying the salt sensitive allelic combination ColColShaSha at the four interacting QTLs, and 124 lines for RILs carrying other allelic at these four QTLs.
(TIF)

Figure S3 **Observed transgression for germination under salt stress in the Sha x Col RIL population.** Germination under 175 mM NaCl 10 days after sowing is presented for 4 RILs and the parental lines.
(TIF)

Figure S4 **LOD trace of MQM mapping for germination under 175 mM NaCl in the Sha x Col RIL population.** LOD trace along the 5 chromosomes of *Arabidopsis thaliana* obtained from MQM mapping analysis (see Materials and Methods) for germination under 175 mM NaCl in the Sha x Col RIL population is presented in red. Markers used as cofactors are indicated with green dots. Marker names are indicated according to their genetic position on each chromosome. The dashed lines indicate the threshold LOD (2.4) determined by permutation test. The position of the peak of QTL1, QTL2, QTL3 and QTL5 is indicated.
(TIF)

Figure S5 **p_value_ trace of Student test performed marker per marker over a subset of 19 selected RILs from the Sha x Col population.** The selected 19 RILs carry the no-germination allelic combination (Col alleles at QTL1 and QTL2 and Sha alleles at QTL5). P_value_ trace is indicated in red and dashed line represents p_value_ threshold of 0.05. Chromosomes are represented by vertical black bars and red lines indicate marker positions.
(TIF)

Figure S6 **Graphical representation of the genotype of RIL173 used for the selection of HIF173_Sha_ and HIF173_Col_.** The 5 Chromosomes of *Arabidopsis thaliana* are represented in vertical bars. Marker names and genetic positions (in cM) are indicated on the left and on the right of each chromosome respectively. For each marker position, the allelic condition is color coded (see legend). The positions of the 4 QTLs are indicated by boxes. The width of each box corresponds to the 2-LOD confidence interval of the QTL (see Supporting Information Table S3).
(TIF)

Table S1 **Origin and responses to salt stresses of 87** ***Arabidopsis thaliana*** **accessions.** Name, Stock number (^N^, NASC stock center (http://arabidopsis.info/); ^A^, ABRC stock center (http://abrc.osu.edu/); ^W^, Wageningen university collection. ^V^, INRA Versailles collection (http://dbsgap.versailles.inra.fr/vnat/)) and country of origin of the 87 selected accessions are reported. In the last 2 columns, reduction in root length in response to 125 mM NaCl (see Materials and Methods) and percentage of germinating seeds which developed viable green seedlings under 200 mM NaCl are given.
(DOC)

Table S2 **Information concerning the RIL populations used for QTL analysis**
(DOC)

Table S3 **Characterization of the QTLs detected in the Sha x Col and Sha x L** ***er*** **RIL populations.** ^a^Marker nearest the highest LOD score; ^b^position in cM at the peak of LOD score; ^c^positive value indicates that Sha alleles increased the trait value; ^d^positions given correspond to 2-LOD confidence intervals.
(DOC)

Table S4 **ANOVA analysis including QTL x QTL terms between the detected germination QTLs in the Sha x Col population**
(DOC)
