# Supplementary figures and images for: Natural Variation in Arabidopsis thaliana Revealed a Genetic Network Controlling Germination Under Salt Stress
Source: PLoS One. 2010 Dec 20;5(12):e15198. doi: 10.1371/journal.pone.0015198 (PMC3004798; doi:10.1371/journal.pone.0015198)

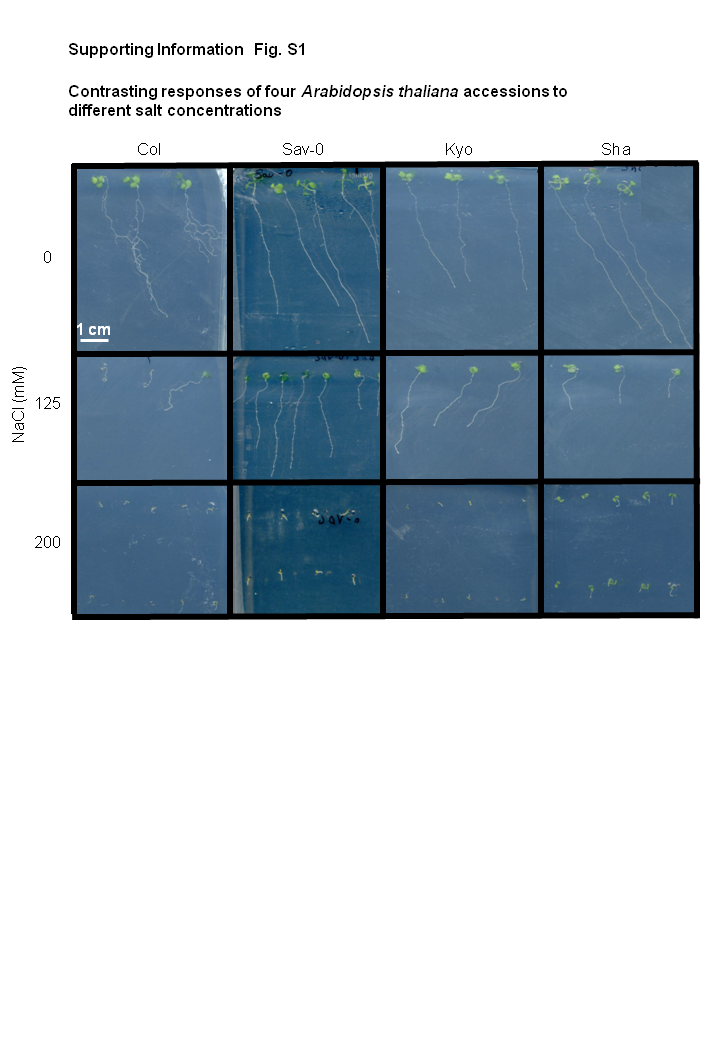

Supplement: Figure S1 — Contrasting responses of four Arabidopsis thaliana accessions to different salt concentrations. Pictures of 10 days old seedlings from four Arabidopsis thaliana accessions (Col, Sav-0, Kyo and Sha – Supporting Information Table S1) grown in agar medium supplemented with 0, 125 and 200 mM NaCl. (TIF) [file pone.0015198.s001.tif]

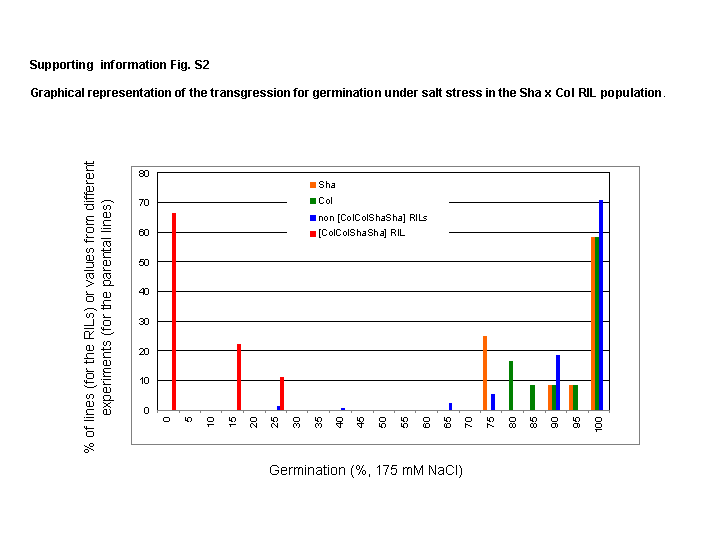

Supplement: Figure S2 — Graphical representation of the transgression for germination under salt stress in the Sha x Col RIL population. Percentage of lines (for the RILs) or values from different experiments (for the parental lines) was plotted against percentage of germination under 175 mM NaCl in each genotypic group. n = 12 different experiments for the parental lines, 9 lines for RILs carrying the salt sensitive allelic combination ColColShaSha at the four interacting QTLs, and 124 lines for RILs carrying other allelic at these four QTLs. (TIF) [file pone.0015198.s002.tif]

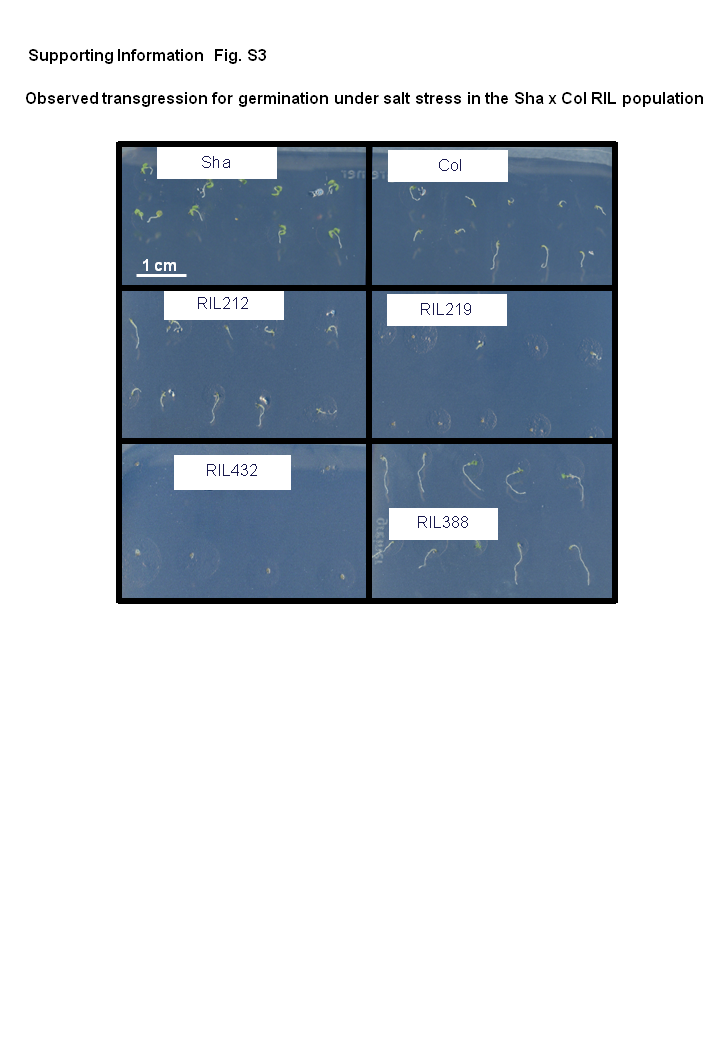

Supplement: Figure S3 — Observed transgression for germination under salt stress in the Sha x Col RIL population. Germination under 175 mM NaCl 10 days after sowing is presented for 4 RILs and the parental lines. (TIF) [file pone.0015198.s003.tif]

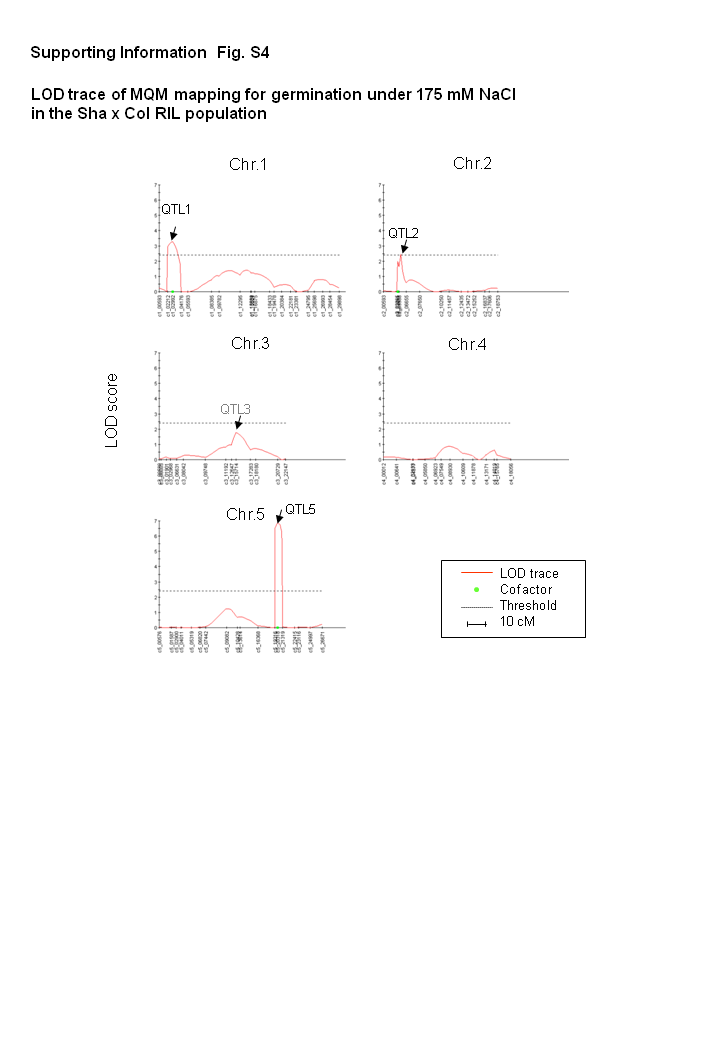

Supplement: Figure S4 — LOD trace of MQM mapping for germination under 175 mM NaCl in the Sha x Col RIL population. LOD trace along the 5 chromosomes of Arabidopsis thaliana obtained from MQM mapping analysis (see Materials and Methods) for germination under 175 mM NaCl in the Sha x Col RIL population is presented in red. Markers used as cofactors are indicated with green dots. Marker names are indicated according to their genetic position on each chromosome. The dashed lines indicate the threshold LOD (2.4) determined by permutation test. The position of the peak of QTL1, QTL2, QTL3 and QTL5 is indicated. (TIF) [file pone.0015198.s004.tif]

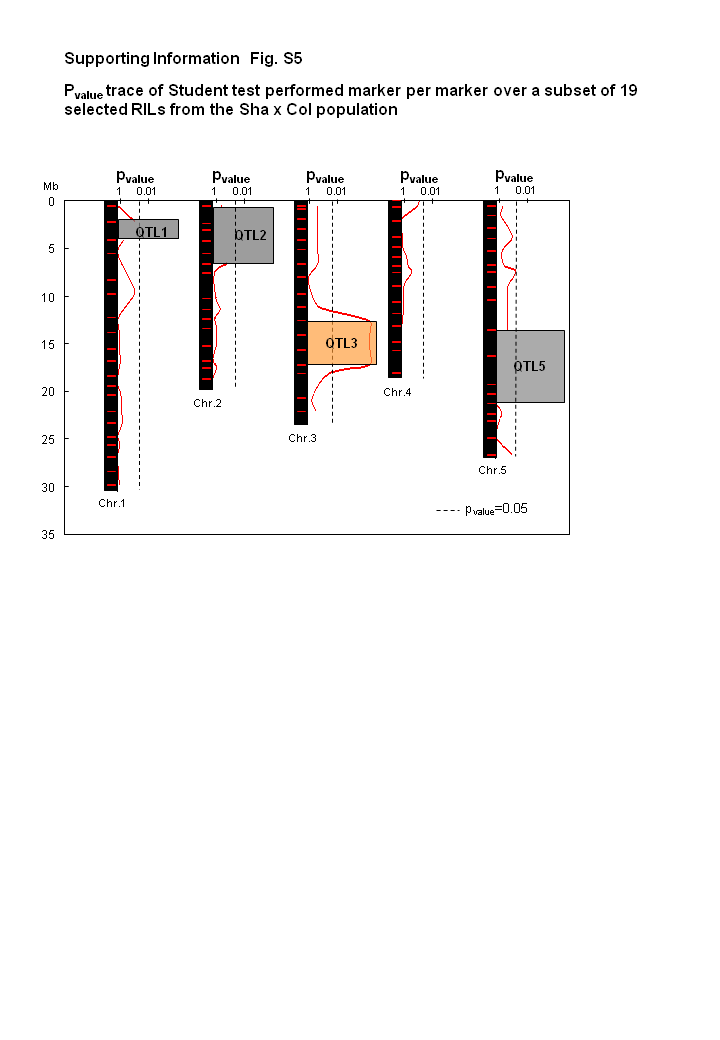

Supplement: Figure S5 — pvalue trace of Student test performed marker per marker over a subset of 19 selected RILs from the Sha x Col population. The selected 19 RILs carry the no-germination allelic combination (Col alleles at QTL1 and QTL2 and Sha alleles at QTL5). Pvalue trace is indicated in red and dashed line represents pvalue threshold of 0.05. Chromosomes are represented by vertical black bars and red lines indicate marker positions. (TIF) [file pone.0015198.s005.tif]

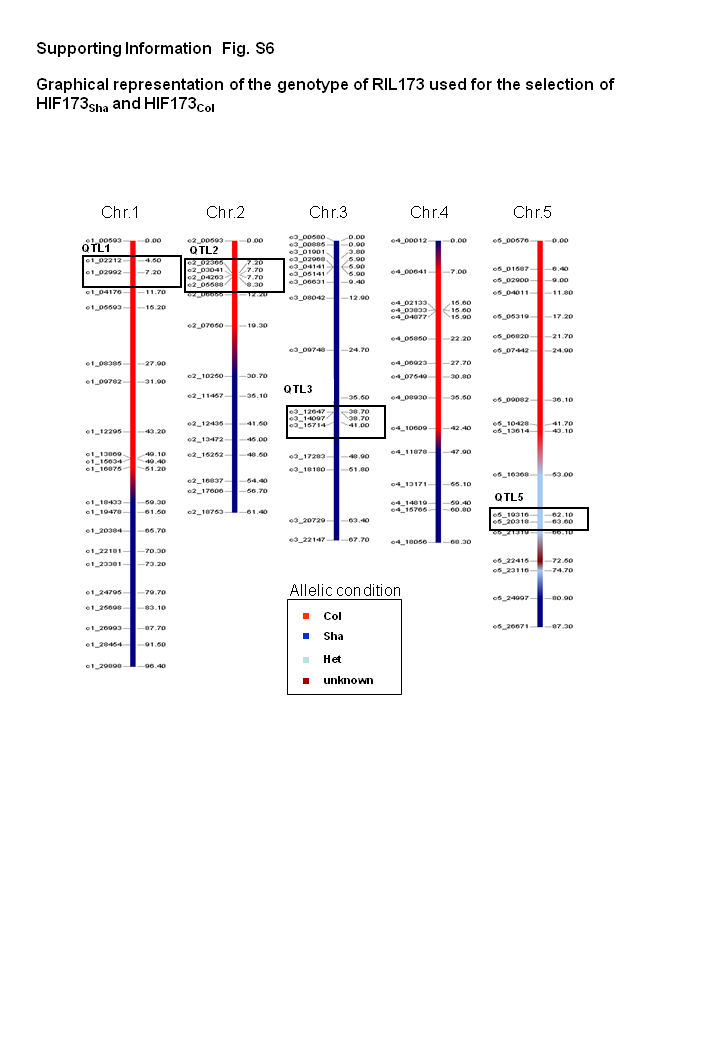

Supplement: Figure S6 — Graphical representation of the genotype of RIL173 used for the selection of HIF173Sha and HIF173Col. The 5 Chromosomes of Arabidopsis thaliana are represented in vertical bars. Marker names and genetic positions (in cM) are indicated on the left and on the right of each chromosome respectively. For each marker position, the allelic condition is color coded (see legend). The positions of the 4 QTLs are indicated by boxes. The width of each box corresponds to the 2-LOD confidence interval of the QTL (see Supporting Information Table S3). (TIF) [file pone.0015198.s006.tif]
