# Supplementary material for: Natural Variation in Arabidopsis thaliana Revealed a Genetic Network Controlling Germination Under Salt Stress
Source: PLoS One. 2010 Dec 20;5(12):e15198. doi: 10.1371/journal.pone.0015198 (PMC3004798; doi:10.1371/journal.pone.0015198)
Supplement: Table S1 — Origin and responses to salt stresses of 87 Arabidopsis thaliana accessions. Name, Stock number (N, NASC stock center (http://arabidopsis.info/); A, ABRC stock center (http://abrc.osu.edu/); W, Wageningen university collection. V, INRA Versailles collection (http://dbsgap.versailles.inra.fr/vnat/)) and country of origin of the 87 selected accessions are reported. In the last 2 columns, reduction in root length in response to 125 mM NaCl (see Materials and Methods) and percentage of germinating seeds which developed viable green seedlings under 200 mM NaCl are given. (DOC) [file pone.0015198.s007.doc]

**Supporting Information Table S1. Origin and responses to salt stresses of 87 *Arabidopsis thaliana* accessions**

| Accession | stock No | Country | response (%, 125 mM NaCl) | Germination and Survival (%, 200 mM NaCl) |
| --- | --- | --- | --- | --- |
| Ag-0 | 901^N^ | France | 100 | 0 |
| Amel-1 | 22526^W^ | Netherlands | 90 | 0 |
| Amel-2 | 22527^W^ | Netherlands | 90 | 0 |
| An-1 | 944^N^ | Belgium | 88 | 0 |
| Bay-0 | 954^N^ | Germany | 80 | 0 |
| Br-0 | 994^N^ | Czech Rep | 72 | 0 |
| Bur-0 | 1028^N^ | Ireland | 45 | 10 |
| C24 | 906^N^ | Portugal | 42 | 30 |
| Cam-4 | 40297^W^ | France | 77 | 0 |
| Can-0 | 1064^N^ | Spain | 93 | 0 |
| Cha-0 | 1068^N^ | Switzerland | 100 | 0 |
| Col-0 | 1092^N^ | Poland | 70 | 0 |
| Ct-1 | 1094^N^ | Italy | 60 | 0 |
| Cvi-0 | 902^N^ | Cape Verde Islands | 82 | 0 |
| Dja-4 | 40399^W^ | Kyrgyzstan | 75 | 0 |
| Eil-0 | 1132^N^ | Germany | 38 | 0 |
| Eri-1 | 22548^N^ | Sweden | 71 | 0 |
| Es-0 | 1144^N^ | Finland | 57 | 10 |
| Est-0 | 1148^N^ | Estonia | 53 | 0 |
| Est-1 | 1150^N^ | Estonia | 63 | 0 |
| Fei-0 | 22645^A^ | Portugal | 67 | 0 |
| Föhr-1 | 40005^W^ | Germany | 70 | 0 |
| Föhr-3 | 40007^W^ | Germany | 100 | 0 |
| Föhr-4 | 40008^W^ | Germany | 79 | 0 |
| Ga-0 | 1180^N^ | Germany | 66 | 0 |
| Grav-1 | 75677^A^ | Netherlands | 80 | 0 |
| Gy-0 | 1216^N^ | France | 46 | 0 |
| Hog | 922^N^ | Tadjikistan | 84 | 20 |
| Jea | 76148^A^ | France | 79 | 0 |
| Kar-2 | 40380^W^ | Kyrgyzstan | 70 | 0 |
| Kas-2 | 28378^A^ | India | 100 | 0 |
| Kin-0 | 22654^A^ | USA | 86 | 0 |
| Kond | 22651^A^ | Tadjikistan | 73 | 0 |
| Kyo-1 | 10372^W^ | Japan | 49 | 0 |
| Kyr-3 | 40409^W^ | Kyrgyzstan | 51 | 0 |
| Kz-1 | 22606^A^ | Kazakhstan | 76 | 0 |
| KZ-13 | 22445^W^ | Kazakhstan | 64 | 0 |
| Kz-9 | 22607^A^ | Kazakhstan | 71 | 0 |
| Ldv-4 | 40271^W^ | France | 77 | 0 |
| Le-1 | [75691](http://www.arabidopsis.org/servlets/TairObject?type=stock&id=3501656731)^A^ | Netherlands | 85 | 0 |
| Le-5 | [75692](http://www.arabidopsis.org/servlets/TairObject?type=stock&id=3501656732)^A^ | Netherlands | 100 | 0 |
| Ler | 1642^N^ | Poland | 77 | 0 |
| Li-5 | 1320^N^ | Germany | 84 | 0 |
| Mog-11 | 40923^W^ | France | 100 | 0 |
| Molde-1 | 40116^W^ | Norway | 100 | 0 |
| Mr-0 | 1372^N^ | Italy | 100 | 0 |
| Mt-0 | 1380^N^ | Lybia | 76 | 0 |
| Mz-0 | 1382^N^ | Germany | 55 | 0 |
| Neo-2 |  | Tadjikistan | 77 | 50 |
| Neo-3 | 539A^V^ | Tadjikistan | 71.5 | 80 |
| Nes-1 | 10041^W^ | Netherlands | 66 | 0 |
| No-0 | 1394^N^ | Germany | 68 | 0 |
| Nok-10 |  | Netherlands | 88 | 0 |
| Nok-11 |  | Netherlands | 78 | 0 |
| Nok-3 | 22643^A^ | Netherlands | 36 | 0 |
| Oerd-2 | 10299^W^ | Netherlands | 66 | 0 |
| Oerd-3 | 10417^W^ | Netherlands | 93 | 0 |
| Oerd-7 | 10418^W^ | Netherlands | 94 | 0 |
| OVliel-1 | 10440^W^ | Netherlands | 81 | 0 |
| OVliel-2 | 10441^W^ | Netherlands | 85 | 0 |
| OVliel-4 | 10443^W^ | Netherlands | 94 | 0 |
| OVliel-ms-1 | 10449^W^ | Netherlands | 76 | 0 |
| OVliel-mw-1 | 10444^W^ | Netherlands | 95 | 0 |
| Pyl-4 | 40425^W^ | France | 91 | 0 |
| Ri-0 | 1492^N^ | Canada-BC | 87 | 0 |
| Sav-0 | 1514^N^ | Czech Rep | 39 | 0 |
| Sf-2 | 1516^N^ | Spain | 62 | 10 |
| Sha | 929^N^ | Tadjikistan | 77 | 100 |
| Sij-1 | 10281^W^ | Uzbekistan | 70 | 40 |
| Sij-4 | 10283^W^ | Uzbekistan | 62 | 0 |
| Sorbo | 931^N^ | Tadjikistan | 81 | 0 |
| Stw-0 | 1358^N^ | Russia | 55 | 0 |
| Sus-3 | 40392^W^ | Kyrgyzstan | 100 | 0 |
| Ts-5 | 1588^N^ | Spain | 90 | 0 |
| Tsu-1 | 1640^N^ | Japan | 72 | 20 |
| Van-0 | 1584^N^ | Canada | 100 | 0 |
| Ovliel-2 | 10441^W^ | Netherlands | 84 | 0 |
| Ws-0 | 1602^N^ | Russia | 58 | 0 |
| Wt-5 | 1612^N^ | Germany | 84 | 50 |
| W-Ter-1 | 10452^W^ | Netherlands | 84 | 0 |
| W-Ter-8 | 10459^W^ | Netherlands | 76 | 0 |
| W-Ter-10 | 10461^W^ | Netherlands | 87 | 0 |
| W-Ter-12 | 10463^W^ | Netherlands | 100 | 0 |
| W-Ter-13 | 10464^W^ | Netherlands | 87 | 0 |
| W-Ter-18 | 10469^W^ | Netherlands | 100 | 0 |
| Yo-0 | 1622^N^ | USA | 91 | 0 |
| Zal-3 | 40406^W^ | Kyrgyzstan | 100 | 0 |

Name, Stock number (^N^, NASC stock center (http://arabidopsis.info/); ^A^, ABRC stock center (http://abrc.osu.edu/); ^W^, Wageningen university collection. ^V^, INRA Versailles collection (<http://dbsgap.versailles.inra.fr/vnat/)>) and country of origin of the 87 selected accessions are reported. In the last 2 columns, reduction in root length in response to 125 mM NaCl (see Materials and Methods) and percentage of germinating seeds which developed viable seedlings under 200 mM NaCl are given.
