# Supplementary material for: Natural Variation in Arabidopsis thaliana Revealed a Genetic Network Controlling Germination Under Salt Stress
Source: PLoS One. 2010 Dec 20;5(12):e15198. doi: 10.1371/journal.pone.0015198 (PMC3004798; doi:10.1371/journal.pone.0015198)
Supplement: Table S2 — Information concerning the RIL populations used for QTL analysis (DOC) [file pone.0015198.s008.doc]

**Supporting Information Table S2. Information concerning the RIL populations used for QTL analysis**

| Population | Number of RILs | Generation | Markers | Reference |
| --- | --- | --- | --- | --- |
| Sha x Col | 164 | F8 | 86 | Simon et al, 2008 |
| Sha x L*er* | 114 | F10 | 76 | Clerkx et al. 2004 |
