# Supplementary material for: Natural Variation in Arabidopsis thaliana Revealed a Genetic Network Controlling Germination Under Salt Stress
Source: PLoS One. 2010 Dec 20;5(12):e15198. doi: 10.1371/journal.pone.0015198 (PMC3004798; doi:10.1371/journal.pone.0015198)
Supplement: Table S3 — Characterization of the QTLs detected in the Sha x Col and Sha x L er RIL populations. aMarker nearest the highest LOD score; bposition in cM at the peak of LOD score; cpositive value indicates that Sha alleles increased the trait value; dpositions given correspond to 2-LOD confidence intervals. (DOC) [file pone.0015198.s009.doc]

**Supporting Information Table S3. Characterization of the QTLs detected in the Sha x Col and Sha x *Ler* RIL populations.**

| **Trait** | **population** | **chromosome** | **Marker^a^** | **Pos (cM)^b^** | **LOD** | **additive effect^c^** | **variance explained (%)** | **total variance explained (%)** | **Confidence interval^d^ (cM)** |
| --- | --- | --- | --- | --- | --- | --- | --- | --- | --- |
| root length 0 mM | Sha x *Ler* | 3 | CHIB | 4 | 2.5 | 0.64 | 8.8 |  | 0-7 |
|  |  | 3 | M3-23 | 29.9 | 2.7 | 0.8 | 9 |  | 26-37 |
|  |  | 3 | F8J2 | 56.2 | 3.5 | -0.94 | 12.4 | 25.6 | 50-64 |
| root length 0 mM | Sha x Col | 4 | c4_00641 | 8 | 3.5 | -0.76 | 5.8 |  | 2-15 |
|  |  | 5 | c5_02900 | 10 | 22.2 | -2.28 | 48.4 | 55.1 | 9-12 |
| root length 125 mM | Sha x *Ler* | 1 | F6D8-94 | 52.7 | 2.6 | -0.3 | 9.3 |  | 48-61 |
|  |  | 2 | T2N18 | 54 | 2.7 | 0.3 | 10 | 20.7 | 49-58 |
| root length 125 mM | Sha x Col | 1 | c1_26993 | 88.7 | 3.9 | 0.32 | 7.5 |  | 83-92 |
|  |  | 2 | c2_07650 | 23.3 | 2.4 | -0.28 | 5.1 |  | 14-31 |
|  |  | 3 | c3_01901 | 2.9 | 3.7 | -0.32 | 7.3 |  | 0-6 |
|  |  | 5 | c5_02900 | 10 | 12.6 | -0.66 | 28.7 | 46.7 | 8-12 |
| response | Sha x *Ler* | 1 | F6D8-94 | 52.7 | 3.5 | 7.02 | 12.4 |  | 49-58 |
|  |  | 3 | M3-32 | 35.1 | 4.8 | 10.98 | 18 |  | 32-41 |
|  |  | 3 | M3-21 | 52.2 | 3.1 | -9.3 | 13.4 | 26.2 | 47-56 |
| response | Sha x Col | 1 | c1_09782 | 35.9 | 3.4 | -6.7 | 12.7 |  | 30-43 |
|  |  | 3 | c3_08855 | 1.9 | 3 | 5.6 | 9.6 | 15.5 | 0-4 |
| germination 175 mM | Sha x *Ler* | 1 | F3F19 | 20.1 | 5.8 | 0.3 | 23.1 |  | 16-24 |
|  |  | 1 | M1-7 | 28.6 | 3.9 | -0.18 | 11.9 |  | 27-32 |
|  |  | 2 | M2-17 | 41.3 | 3.3 | 0.12 | 9.9 | 30.4 | 37-43 |
| Germination 175 mM | Sha x Col | 1 (QTL1) | c1_02992 | 7.2 | 3.3 | 0.14 | 7.8 |  | 4-10 |
|  |  | 2 (QTL2) | c2_04263 | 9.3 | 2.5 | 0.1 | 4.6 |  | 7-11 |
|  |  | 5 (QTL5) | c5_20318 | 63.6 | 6.8 | -0.22 | 17.3 | 29.8 | 62-66 |

^a^Marker nearest the highest LOD score; ^b^position in cM at the peak of LOD score; ^c^positive value indicates that Sha alleles increased the trait value; ^d^positions given correspond to 2-LOD confidence intervals.
