# Supplementary material for: Natural Variation in Arabidopsis thaliana Revealed a Genetic Network Controlling Germination Under Salt Stress
Source: PLoS One. 2010 Dec 20;5(12):e15198. doi: 10.1371/journal.pone.0015198 (PMC3004798; doi:10.1371/journal.pone.0015198)
Supplement: Table S4 — ANOVA analysis including QTL x QTL terms between the detected germination QTLs in the Sha x Col population (DOC) [file pone.0015198.s010.doc]

**Supporting Information Table S4. ANOVA including only main effects or main effect and QTL x QTL terms between the detected germination QTLs in the Sha x Col population.**

(A) Model including only the four main effect QTLs for germination under 175 mM NaCl

| Source | Type III sum of Squares | df | Mean Square | F | Sig, | r^2^ |
| --- | --- | --- | --- | --- | --- | --- |
| QTL1 | 0,646 | 1 | 0,646 | 13,88 | 0 | 9,9 |
| QTL2 | 0,429 | 1 | 0,429 | 9,226 | 0,003 | 6,8 |
| QTL3 | 0,149 | 1 | 0,149 | 3,193 | 0,076 | 2,5 |
| QTL5 | 1,682 | 1 | 1,682 | 36,155 | 0 | 22,3 |
| Error | 5,862 | 126 | 0,47 |  |  |  |
| Total | 8,813 | 130 |  |  |  | 33,5 |

(B) Model including main effect QTLs for germination under 175 mM NaCl and epistatic interactions between them

| Source | Type III sum of Squares | df | Mean Square | F | Sig, | r^2^ |
| --- | --- | --- | --- | --- | --- | --- |
| QTL1*QTL2*QTL3*QTL5 | 3,038 | 11 | 0,276 | 11,245 | 0 | 51,8 |
| QTL1 | ,454 | 1 | 0,454 | 18,470 | 0 | 13,8 |
| QTL2 | ,598 | 1 | 0,598 | 24,342 | 0 | 17,5 |
| QTL3 | ,227 | 1 | 0,227 | 9,234 | 0,003 | 7,4 |
| QTL5 | ,908 | 1 | 0,908 | 36,990 | 0 | 24,3 |
| Error | 2,824 | 115 | 0,025 |  |  |  |
| Total | 8,183 | 130 |  |  |  | 68 |
